# WIP and WICH/WIRE co-ordinately control invadopodium formation and maturation in human breast cancer cell invasion

**DOI:** 10.1038/srep23590

**Published:** 2016-03-24

**Authors:** Esther García, Chiara Ragazzini, Xinzi Yu, Elena Cuesta-García, Jorge Bernardino de la Serna, Tobias Zech, David Sarrió, Laura M. Machesky, Inés M. Antón

**Affiliations:** 1 Centro Nacional de Biotecnología (CNB-CSIC), Madrid, Spain; 2 The Beatson Institute for Cancer Research, Glasgow, UK; 3 Science and Technology Facilities Council, Rutherford Appleton Laboratory, Central Laser Facility, Research Complex at Harwell, Harwell-Oxford, UK; 4 Universidad Autónoma de Madrid, Madrid, Spain

## Abstract

Cancer cells form actin-rich degradative protrusions (invasive pseudopods and invadopodia), which allows their efficient dispersal during metastasis. Using biochemical and advanced imaging approaches, we demonstrate that the N-WASP-interactors WIP and WICH/WIRE play non-redundant roles in cancer cell invasion. WIP interacts with N-WASP and cortactin and is essential for invadopodium assembly, whereas WICH/WIRE regulates N-WASP activation to control invadopodium maturation and degradative activity. Our data also show that Nck interaction with WIP and WICH/WIRE modulates invadopodium maturation; changes in WIP and WICH/WIRE levels induce differential distribution of Nck. We show that WIP can replace WICH/WIRE functions and that elevated WIP levels correlate with high invasiveness. These findings identify a role for WICH/WIRE in invasiveness and highlight WIP as a hub for signaling molecule recruitment during invadopodium generation and cancer progression, as well as a potential diagnostic biomarker and an optimal target for therapeutic approaches.

Metastasis is a multistep process wherein cancer cells separate from the primary tumor, invade the surrounding extracellular matrix (ECM), and disperse throughout the body via blood or lymphatic systems, to reach distant tissues where they proliferate and are established as a secondary tumor^1^,^2^. Metastasis is responsible for ~90% of cancer mortality; it involves several signaling cascades as well as actin cytoskeleton reorganization^3^,^4^. Matrix-cell interactions drive the first steps of cancer progression by adapting internal signaling pathways in response to external stimuli from the ECM^5^. Some of these signals regulate cell invasiveness and motility via matrix degradation, through development of invasive pseudopods such as invadopodia^6^,^7^.

Invadopodia are actin-rich cancer cell protrusions with proteolytic activity; they concentrate adhesion and scaffolding proteins, actin-nucleating factors, kinases and metalloproteinases^8^,^9^. These structures depend on regulated Src kinase activity^10^,^11^,^12^ and on actin-regulating proteins such as the Arp2/3 complex, cortactin, the adaptor protein Nck^11^,^13^,^14^,^15^,^16^ and N-WASP (neural-Wiskott-Aldrich syndrome protein), an important contributor to cancer invasion *in vitro* and *in vivo*^16^,^17^,^18^. N-WASP and WIP (WASP-interacting protein) localize at invasive protrusions^16^,^19^,^20^,^21^, and their interaction might promote invadopodium formation^16^. Invadopodia assemble sequentially^11^,^15^, starting with formation of a precursor that matures into a degradation-competent invadopodium. A more detailed model summarizes the molecular mechanisms that underlie precursor initiation, stabilization, and maturation into a functional invadopodium: during invadopodium precursor initiation, cortactin (or cortactin/actin complex) acts as a scaffold that captures cofilin and N-WASP to form the initial precursor core. Nck recruitment to invadopodia then activates actin polymerization^11^, followed by Tks5 recruitment and precursor stabilization^22^. WIP is an indispensible component of invadopodia^20^,^23^, but the precise timing of its role in invadopodium lifetime (initiation, stabilization or maturation) is not fully known. We know little of the separate or redundant roles in cancer cell invasion of other N-WASP-interacting WIP family members^24^,^25^,^26^, CR16 (corticosteroids and regional expression 16) and WICH/WIRE (WIP-CR16 homologous/WIP-related).

As CR16 expression is restricted mostly to brain and testis^25^, here we determined the contribution of WIP and WIRE to breast cancer cell (BCC) invasiveness and the role of these proteins in cytoskeletal organization of the invasion machinery. Using diverse approaches to examine cell motility (2D and 3D invasion systems) and advanced fluorescence imaging, we demonstrate that WIP and WIRE have dissimilar expression patterns in invasive and non-invasive BCC lines. These proteins also have non-redundant functions in invading cells, as both are necessary for efficient cell dissemination throughout the ECM.

## Results

### WIP and WIRE are necessary for efficient cell invasion in 3D matrices and control different stages of cell invasion

**Figure 1:**
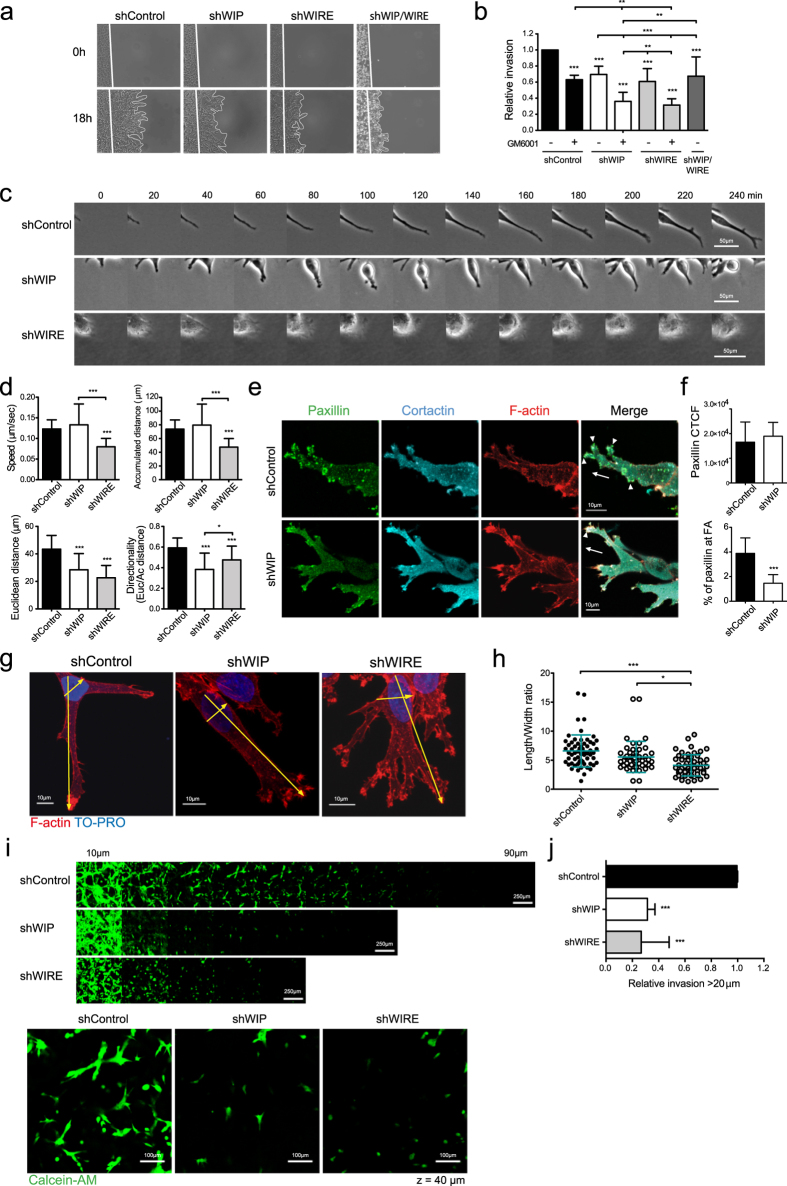
WIP and WIRE are necessary for efficient degradation and migration in 3D matrices (circular invasion assays, CIA). (**a**) shControl, shWIP, shWIRE and double shWIP/WIRE knock-down MDA-MB-231 cells were cultured with Matrigel (25 μM GM6001 or carrier, DMSO). White lines in time-lapse microscopy images indicate the cell front at t =  0 and 18 h. (**b**) The cell-free area was measured and relative invasion was normalized to invaded area in control samples. (**c**) Time-lapse images of cell protrusions. Bars: 50 μm. (**d**) Quantification of speed, accumulated and Euclidean distances, and directionality of leading cells invading Matrigel. (**e**) Cells were incubated (24 h) before fixing in 4% paraformaldehyde and immunofluorescence (IF) analyses (cells stained for paxillin (green), cortactin (cyan) and F-actin (red)). Arrows indicate the direction of invading cells; arrowheads show invasive protrusions where paxillin, cortactin and F-actin localize. Bars: 10 μm. (**f**) Quantification of cell total corrected fluorescence (CTCF, top) and of the area occupied by paxillin-positive puncta compared to total paxillin (bottom) (see Methods). (**g**) IF images of cells invading Matrigel, stained for F-actin (red) and nuclei (TO-PRO, blue). Bars: 10 μm. (**h**) Quantification of length/width ratio of leading cells invading Matrigel. Length was calculated as distance from the most distal protruding tip of the cell to the opposite base of the nucleus. Width was measured as the widest cell distance across the nucleus. Each dot represents a single cell. (**i**) Stably infected shControl, shWIP or shWIRE MDA-MB-231 cells were allowed to invade Matrigel plugs in an inverted invasion assay. Bars: 250 μm. After 4 d invasion, cells were stained with live marker calcein-AM (4 μM, 1 h, 37 °C) and serial optical sections (10 μm intervals) were acquired. Magnified images from z = 40 μm sections are shown (bottom). Bars: 100 μm. (**j**) Cell invasion was quantified as cell-covered area >20 μm and then normalized to control values. Data show mean ± SD of three independent experiments. *p < 0.05, **p < 0.01; ***p < 0.001 by 2-way ANOVA and Bonferroni’s post-hoc test (**b**), by 1-way ANOVA and Tukey’s post-hoc test (**d,h,j**) or Student’s t-test (**f**).

WIP is necessary for invadopodium-mediated cell invasion^23^, but little is known of how it contributes to the underlying mechanism. This, and previous studies suggesting the need for N-WASP/WIP interaction to mediate cancer cell invasion^16^,^18^ prompted us to examine the contribution of WIP and WIRE (another N-WASP-binding member of the family) to invasion in 3D/physiological matrices. Since WIP and WIRE are strongly expressed in BCC, we generated stable WIP- and WIRE-deficient MDA-MB-231 cells by expressing shRNAs via lentiviral infection. After testing five independent shRNAs for each protein, we selected two each that reproducibly depleted WIP by 80–90% or WIRE by 75–90% (Supplementary Fig. 1a,b). Here we show representative results obtained with one of each shRNA. In a circular invasion assay (CIA)^21^,^27^, WIP and WIRE depletion reduced cancer cell invasion through Matrigel by 30–40% (Fig. 1a,b). Invasive motility in CIA is dependent on ECM remodeling^18^,^21^. Inhibition of matrix metalloproteinase (MMP) activity using the specific inhibitor GM6001 reduced control cell invasion by 40% (Fig. 1a,b), which was further decreased by GM6001 treatment of WIP- or WIRE-depleted cells (>60%; Fig. 1b). These results indicate that WIP and WIRE are necessary for invasive migration.

To understand how WIP and WIRE mediate invasion, we focused on single-cell events and analyzed differences in leading cell penetration of Matrigel. Whereas control cells formed large, persistent protrusions, WIP depletion promoted formation of unstable structures that protruded and retracted repeatedly, which significantly impaired cell movement and reduced directionality (Fig. 1c,d). These observations suggested that WIP affects adhesion in transformed cells. We therefore examined localization of the focal adhesion (FA) marker paxillin, and found altered distribution in WIP-deficient cells (Fig. 1e,f). WIRE-deficient cells showed decreased persistence as in WIP-deficient cells, as well as reduced speed (Fig. 1c,d). In addition to matrix degradation, WIP and WIRE thus have a role in adhesion and migration. F-actin staining analysis indicated that, compared to WIP-deficient cells, WIRE-deficient cells formed more complex and branched protrusions, but that these were shorter. Length/width ratio analyses showed that WIRE-deficient cells were less elongated than controls (Fig. 1g,h). Results were similar in inverse invasion assays, in which cells invaded thick Matrigel plugs in response to a chemical gradient. WIP- and WIRE-deficient cells invaded less than controls, and WIRE-deficient cells were less polarized, with a more rounded morphology (Fig. 1i,j).

**Figure 2:**
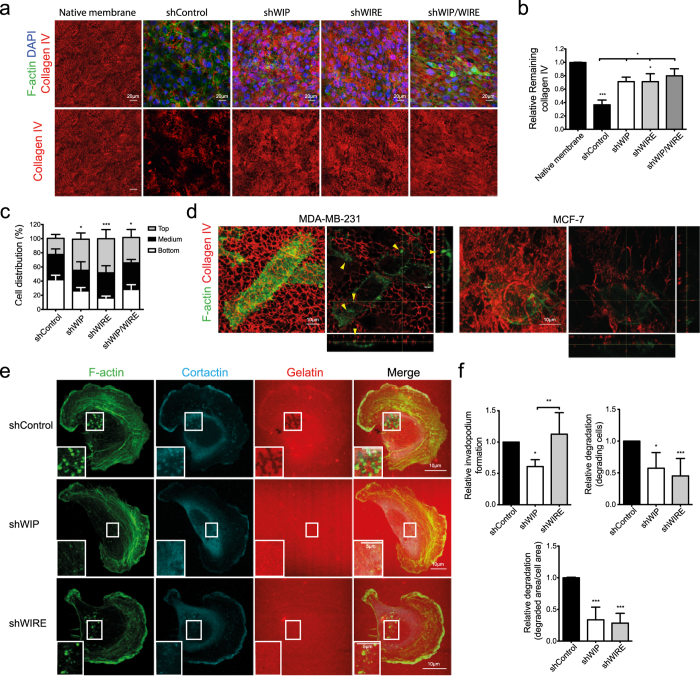
WIP and WIRE are necessary for invadopodium-mediated degradation. (**a**) Stably infected MDA-MB-231 cells (shControl, shWIP, shWIRE and double shWIP/WIRE knock-down) were cultured on mouse peritoneal basement membrane (BM) (4 d), fixed, and stained for mouse type IV collagen (red), nuclei (DAPI, blue) and F-actin (green), and visualized by confocal microscopy. Serial optical sections were captured at 2-μm intervals. Bars: 20 μm. (**b**) Remaining type IV collagen in BM was measured in Z-projections and normalized to native membrane levels. (**c**) Cell distribution across the BM was calculated as the percentage of cells located at three different sectors of the stack: on top (grey), embedded in (black) and beneath the BM (white). (**d**) MDA-MB-231 or MCF-7 cells were cultured on mouse peritoneal BM (24 h), fixed and stained as in (**a**). Bars: 10 μm. Arrowheads indicate invadopodia. (**e**) Stably infected shControl, shWIP or shWIRE MDA-MB-231 cells were plated on rhodamine-gelatin-coated glass coverslips (red, 5 h), fixed and stained for F-actin (green) and cortactin (cyan). Bars: 10 μm (insets, 5 μm). (**f**) Quantification of invadopodium-forming cells (number of cells that form invadopodia), degrading cells (number of cells that degrade gelatin) and degraded area (calculated as degraded area/cell area) were normalized to control values. Data show mean ± SD of at least three independent experiments (N ≥ 75 cells/condition). *p < 0.05, **p < 0.01, ***p < 0.001 by 1-way ANOVA and Tukey’s post-hoc test (**b** and degraded area measurements in (**f**) or Chi-square test (**c** and **f**, for invadopodium-forming and degrading cells).

To test the WIP/WIRE role in remodeling of native crosslinked collagen matrix, we analyzed cell capacity to degrade and invade native mouse peritoneal basement membrane (BM)^28^,^29^. After 4 days incubation, control cells degraded most type IV collagen fibers, seen as a reduction in the collagen IV-specific signal found in BM, but WIP-, WIRE- or WIP/WIRE-depleted cells barely degraded the fibers (Fig. 2a,b). We also analyzed cell distribution and found that 40% of control cells crossed the BM whereas cells lacking WIP, WIRE or WIP/WIRE remained mainly atop the membrane (Fig. 2c). This suggests that the invasion defect is not due solely to migration defects, but that WIP and WIRE help to mediate matrix proteolysis and cell movement.

In contrast to luminal MCF-7, MDA-MB-231 cells develop WIP-bearing invadopodia^20^ to invade the BM and the ECM (Fig. 2d). To quantify invadopodium number and degradation accurately, we cultured WIP- and WIRE-deficient cells on fluorescent gelatin-coated glass coverslips. Whereas control cells formed invadopodia (F-actin- and cortactin-positive dots) and degraded the matrix (dark areas in the gelatin), a significant fraction of WIP-deficient cells was unable to form invadopodia and degrade the gelatin (Fig. 2e,f); the cells able to form invadopodia degraded less gelatin than controls (Fig. 2f). WIRE-deficient cells formed invadopodium-like structures (F-actin- and cortactin-positive) in numbers equivalent to control cells, but as in BM experiments, matrix degradation was impaired (Fig. 2e,f).

WIP and WIRE are thus necessary for matrix degradation and cell migration in diverse matrices, but their contribution to invasion differs, as WIP is necessary for invadopodium formation, whereas WIRE is needed for protrusion maturation.

### WIP and WIRE levels are regulated interdependently

**Figure 3:**
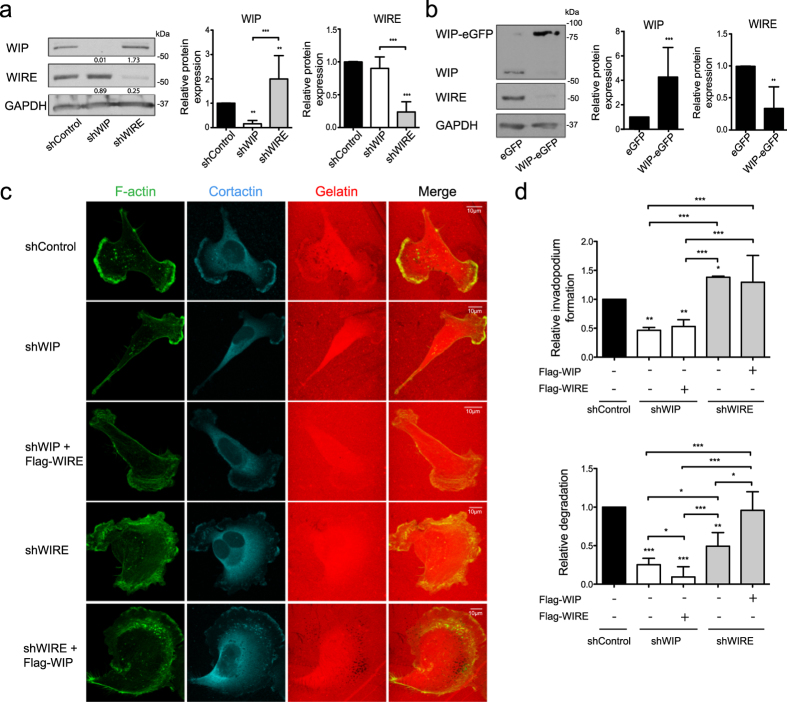
Overexpression of WIP overcomes WIRE deficiency. (**a**) WIP, WIRE and GAPDH levels were analyzed by WB in shControl, shWIP or shWIRE soluble MDA-MB-231 cell lysates (left). Quantification of WB experiments is shown (right). (**b**) Soluble lysates from MDA-MB-231 transiently overexpressing eGFP or WIP-eGFP, WIP and WIRE levels were analyzed by WB (left) and relative expression was quantified using GAPDH expression as a control (right). (**c**) Cells overexpressing Flag-WIRE or Flag-WIP in WIP- and WIRE-deficient cells were allowed to invade rhodamine-gelatin (red, 5 h), fixed in 4% PFA, and stained for F-actin (green) and cortactin (cyan). Bars: 10 μm. (**d**) Quantification of relative invadopodium formation (number of cells that form invadopodia) and degradation (number of cells that degrade gelatin) normalized to control values. Data show mean ± SD of at least three independent experiments (N ≥ 70 cells/condition). *p < 0.05, **p < 0.01, ***p < 0.001 by 1-way ANOVA and Tukey’s post-hoc test (**a**), Student’s *t*-test (**b**) or Chi-square test (**d**).

Since WIP and WIRE are both expressed strongly in BCC and are involved in efficient invasion by MDA-MB-231 cells, we tested whether WIP/WIRE expression is controlled by a compensatory mechanism. Whereas WIP-deficient cells showed no changes in WIRE expression, WIRE silencing significantly increased endogenous WIP; conversely, WIP-eGFP overexpression reduced endogenous WIRE levels (Fig. 3a,b). Although WIRE-deficient cells overexpress WIP endogenously, their degradative ability remained reduced. We thus tested whether exogenous WIP expression increased cell invasiveness, and found that WIP overexpression rescued the gelatin-degrading ability of WIRE-deficient invadopodia, whereas WIRE overexpression did not rescue WIP depletion (Fig. 3c,d). These results indicate complex regulation between WIP and WIRE expression in MDA-MB-231 BCC, in which low WIRE levels correlate with high WIP levels that are insufficient to fully replace WIRE function in invadopodium maturation.

### WIRE regulates N-WASP activation during invadopodium formation

**Figure 4:**
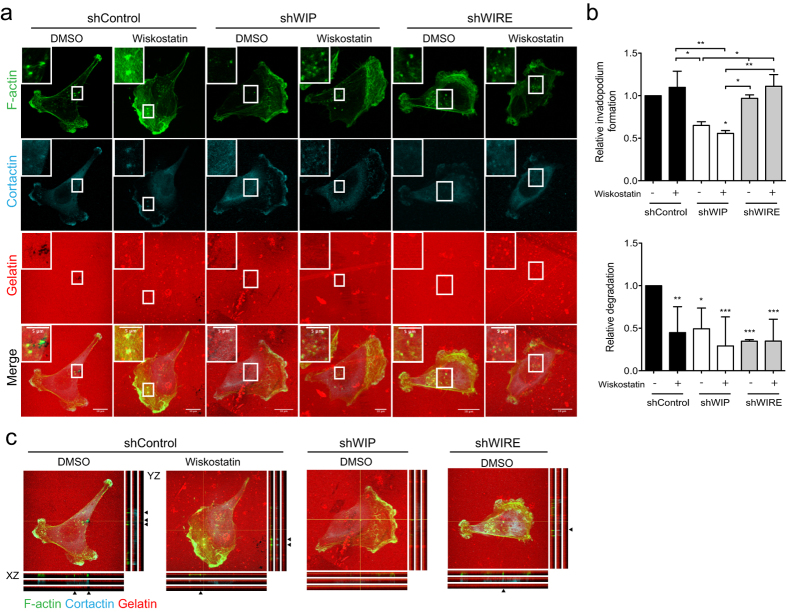
Inhibition of N-WASP activation mimics the lack of WIRE. shControl, shWIP and shWIRE MDA-MB-231 cells were allowed to invade rhodamine-gelatin (red) in the presence of vehicle or 2 μM wiskostatin (3 h). (**a**) Cells were fixed and stained for F-actin (green) and cortactin (cyan). Bars: 10 μm (insets, 5 μm). (**b**) Quantification of relative invadopodium-forming cells (number of cells that form invadopodia) and degrading cells (number of cells that degrade gelatin) normalized to shControl values. Data show mean ± SD of at least three independent experiments (N ≥ 98 cells/condition). *p < 0.05, **p < 0.01, ***p < 0.001, Chi-square test. (**c**) Orthogonal views of shControl (treated or not with wiskostatin), shWIP and shWIRE cells shown in (**a**); arrows indicate invadopodia (F-actin- and cortactin-positive protrusions).

WIP and WIRE interact directly with N-WASP to regulate its activation^24^,^26^,^30^. N-WASP is needed for invadopodium development^17^,^18^, and its interaction with WIP could be central in controlling this process^16^. As WIRE is also expressed in MDA-MB-231 cells and is necessary for matrix degradation (Fig. 2), we hypothesized that WIP, WIRE or both proteins regulate N-WASP activation during invadopodium development. Control cells treated with the N-WASP inhibitor wiskostatin formed invadopodia but did not degrade gelatin, thus mimicking the defects observed in WIRE-deficient but not in WIP-deficient cells (Fig. 4). To determine whether WIP or WIRE regulate N-WASP activation, we added wiskostatin to WIP- or WIRE-deficient cells and observed no additional effect (Fig. 4a,b). WIP deficiency prevented invadopodium formation that was unaltered by wiskostatin treatment, and wiskostatin treatment of control cells reduced gelatin degradation equivalent to WIRE deficiency. These findings indicate that WIP acts upstream of N-WASP activation, whereas WIRE and N-WASP have similar roles during invadopodium development.

### WIP binding to cortactin and N-WASP are necessary for invadopodium formation whereas Nck binding negatively regulates invadopodium maturation

Although N-WASP/WIP interaction might be essential for invadopodium-mediated invasion^16^, our data suggest that WIRE is the main WIP family protein responsible for N-WASP activation and invadopodium maturation. Other WIP-interacting proteins such as Nck and cortactin are reported to be key components of invadopodium activity; cortactin recruitment of the adaptor Nck is necessary for invadopodium maturation^11^,^16^, and Nck interaction with WIP and N-WASP promotes actin polymerization^31^. How these interactions contribute to invadopodium-mediated invasion nonetheless remains unclear.

**Figure 5:**
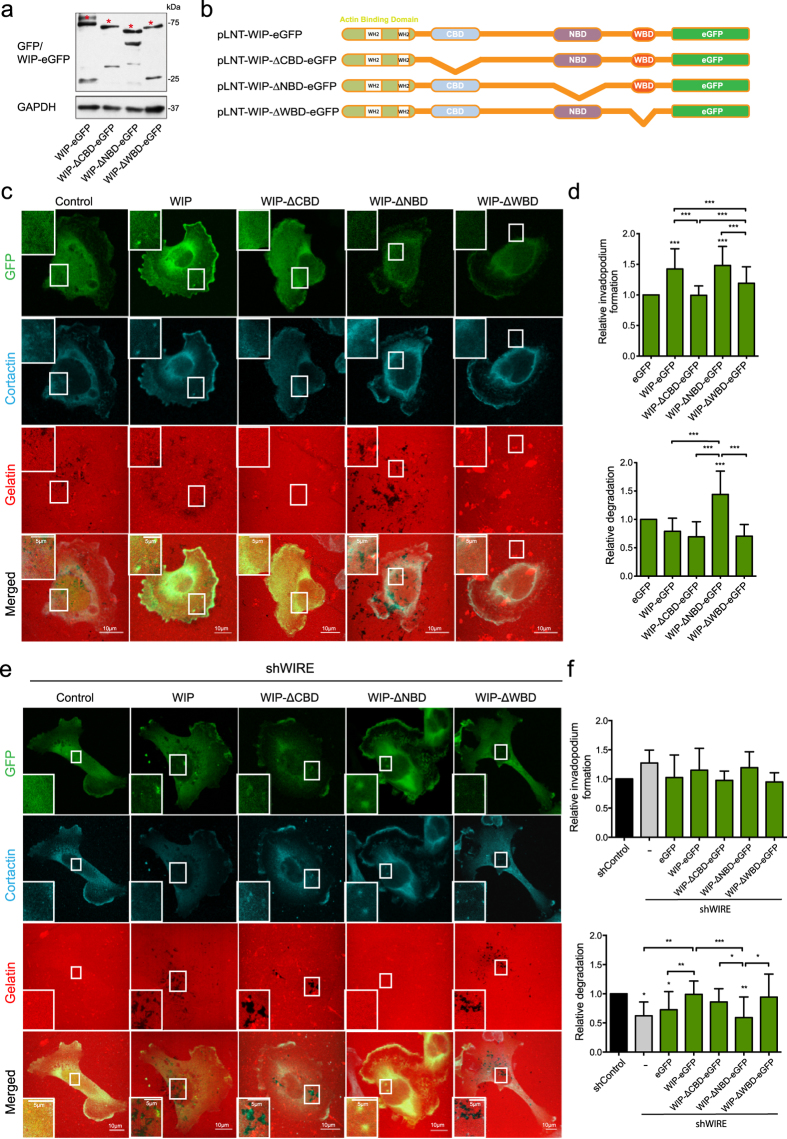
Nck binding to WIP negatively regulates invadopodium maturation. (**a**) WB image shows eGFP fusions (asterisks) and GAPDH expression in which cell lysates were examined using anti-GFP and -GAPDH antibodies. (**b**) Scheme of full length WIP and WIP deletion mutants lacking the binding domains for cortactin (ΔCBD), Nck (ΔNBD) or N-WASP (ΔWBD). (**c**) MDA-MB-231 cells overexpressing WIP or WIP deletion mutants (eGFP, green) ΔCBD, ΔNBD and ΔWBD were plated on rhodamine-gelatin (red, 5 h), fixed and stained for cortactin (cyan). Bars: 10 μm (insets, 5 μm). (**d**) Quantification of invadopodium formation and degradation normalized to eGFP-expressing cell control values. (**e**) Confocal images of shWIRE eGFP-expressing MDA-MB-231 cells (green; control, WIP, ΔCBD, ΔNBD and ΔWBD) and co-stained for cortactin (cyan). Bars: 10 μm (insets, 5 μm). (**f**) Quantification of relative invadopodium formation (number of cells that form invadopodia) and degradation (number of cells that degrade gelatin) normalized to control values. Data show mean ± SD of at least three independent experiments (N ≥ 70 cells/condition). *p < 0.05, **p<0.01, ***p < 0.001 by Chi-square test.

WIP overexpression can compensate for the lack of WIRE and fully rescue invadopodium activity (Fig. 3). To determine how WIP controls invadopodium formation and replaces WIRE functions in MDA-MB-231 cells, we expressed full-length WIP and deletion mutants that lack binding domains to cortactin (WIP-ΔCBD)^32^, Nck (WIP-ΔNBD)^33^ or N-WASP (WIP-ΔWBD)^33^,^34^ (Fig. 5a,b). Overexpression of full-length WIP enhanced in-vadopodium formation, while gelatin degradation remained unaffected; WIP-ΔNBD-expressing cells showed increased invadopodium formation and maturation, whereas WIP-ΔCBD and WIP-ΔWBD did not promote invadopodium formation (Fig. 5c,d and Supplementary Fig. 2a). Nck binding to WIP therefore reduces invadopodium-mediated degradation, indicating a repressive role for Nck.

WIRE-deficient cells that overexpressed WIP-eGFP, WIP-ΔCBD or WIP-ΔWBD regained the gelatin-degrading ability, but not those that expressed WIP-ΔNBD (Fig. 5e,f and Supplementary Fig. 2b). Control cells that expressed WIP-ΔNBD degraded more gelatin than WIP-eGFP-, WIP-ΔCBD- or WIP-ΔWBD-expressing controls. The contrasting phenotypes in control and WIRE-deficient cells with impaired WIP binding to Nck indicate that the presence of Nck in the N-WASP/WIP or N-WASP/WIRE complex defines the invadopodium maturation stage.

### Nck subcellular distribution is WIP- and WIRE-dependent

**Figure 6:**
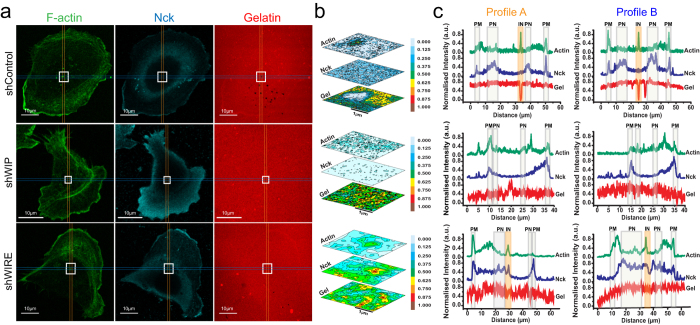
Nck is differentially distributed in the absence of WIP or WIRE. shControl, shWIP and shWIRE MDA-MB-231 cells were allowed to invade rhodamine-gelatin (red, 5 h). (**a**) Cells were fixed and stained for F-actin (green) and Nck (cyan). Bars: 10 μm. (**b**) Normalized contour map from insets in (**a**), dark tones indicate high protein expression and light tones, low expression. (**c**) Profiles of fluorescence intensity of F-actin (green), Nck (blue) and gelatin (red). Profiles were traced in areas containing invadopodia (when present) and represent average measurements of a 10-pixel range. PM =  plasma membrane, PN =  perinuclear, IN =  invadopodium.

To define whether Nck location determines its invadopodium-related activity, we examined Nck distribution in gelatin-plated cells. In control cells, Nck accumulated mainly in perinuclear areas and the leading edge (lamellipodium) (Fig. 6a,c) but not in invadopodium areas (Fig. 6b,c). In WIP-deficient cells, Nck was not observed in central cell areas, suggesting displacement to the periphery (Fig. 6a–c). WIRE-deficient cells showed less Nck in lamellipodia (Fig. 6c); it was found in nuclear and perinuclear regions and around invadopodia (Fig. 6a–c). The data suggest that the WIP/cortactin/N-WASP complex regulates Nck subcellular distribution and that absence of WIRE retains Nck in this complex in the vicinity of the invadopodium, preventing full maturation.

### WIP is expressed strongly in invasive basal-B BCC

**Figure 7:**
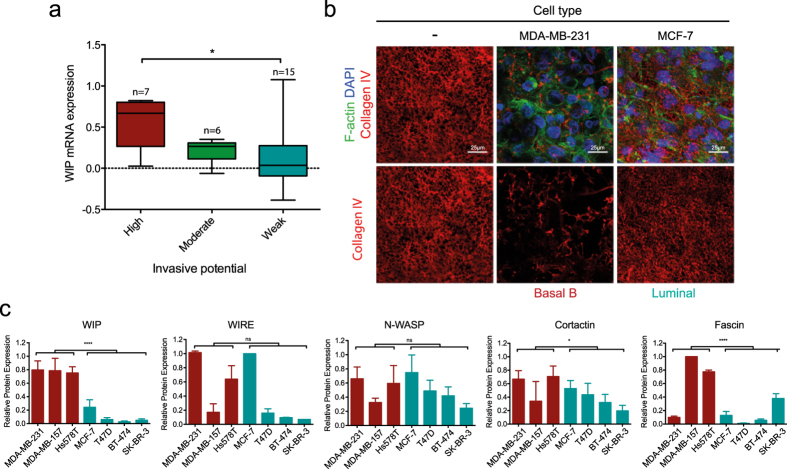
WIP is strongly expressed in invasive basal-B BCC. (**a**) Relationship between WIP mRNA levels and the invasive behavior of breast cancer cells (BCC). Microarray data for WIP gene expression were retrieved from two reports^35^,^36^ and BCC lines were grouped according to their invasive potential, as described^35^. (**b**) MDA-MB-231 and MCF-7 cells were cultured on mouse peritoneal BM (4 d). After fixing in 4% PFA, samples were stained for IF for mouse type IV collagen (red), F-actin (green) and nuclei (DAPI, blue) and visualized by confocal microscopy. Bars: 25 μm. (**c**) Lysates of basal-B (red) and luminal cells (green) were analyzed by WB using anti-WIP, -WIRE, -N-WASP, -cortactin and -fascin antibodies, with GAPDH expression as control (not shown). Protein expression values were normalized to the highest value in each graph. Data show mean ± SD of at least three independent experiments. ns, not significant; *p < 0.05, ****p < 0.0001 by 2-way ANOVA.

Using microarray gene expression data for BCC lines^35^, we analyzed WIP correlation to BCC invasive behavior; highly invasive cell lines showed significantly higher WIP mRNA levels than weakly invasive cells (Fig. 7a and Supplementary Fig. 1c). WIP overexpression also correlated positively with the basal-B subtype (Supplementary Fig. 1d^36^) associated with invasive behavior *in vitro* and *in vivo* compared to less-invasive basal-A and luminal BCC^37^,^38^. To validate the association of WIP overexpression with the invasive behavior of these cells in a more physiological system, we compared the ability of invasive MDA-MB-231 (basal-B) and poorly invasive MCF7 (luminal) cell lines to remodel the ECM on mouse peritoneal BM (Fig. 7b). After 4 days incubation, BM on which MDA-MB-231 cells were cultured showed less remaining type IV collagen (indicating membrane degradation) than those cultured with MCF-7 cells, which maintained nearly intact type IV collagen fibers (Fig. 7b).

In western blot analysis, we confirmed that as for mRNA expression, WIP protein levels were significantly higher in basal-B than in luminal human cells (Fig. 7c), whereas WIRE levels varied and did not correlate with BCC grouping. We analyzed levels of other invadopodium-related proteins such as N-WASP, cortactin and fascin, and found significant differences in cortactin and fascin expression between basal-B and luminal cells (Fig. 7c), which confirmed previous data^39^,^40^. These results suggest that WIP, cortactin and fascin levels correlate with the invasive behavior of BCC, whereas those of WIRE and N-WASP do not. Of the proteins studied, only WIP levels were high in all basal-B cell lines and low in all luminal cell lines analyzed, which highlights its potential as a biomarker for aggressiveness in human breast tumors.

## Discussion

Using biochemical and advanced cellular approaches that mimic *in vivo* tumor invasion conditions, we establish how WIP and WIRE contribute to BCC invasiveness through coordinated roles. We show that WIP is necessary for the assembly of invasive protrusions, whereas WIRE regulates their maturation, which leads to matrix degradation. During invadopodium maturation, Nck can impair or promote ECM degradation, depending on its interaction with WIP/N-WASP or WIRE/N-WASP complexes. Given its high levels in invasive BCC and its ability to overcome WIRE deficiency, we propose WIP as a potential therapeutic target for treatment of metastatic cancer and as a prognostic marker for breast cancer patients.

In MTLn3 adenocarcinoma cells, expression of either N-WASP shRNA or a dominant negative form of N-WASP produces a markedly decreased cellular ability to form invadopodia and degrade ECM^17^. In our cell system (MDA-MB-231), N-WASP inhibition by wiskostatin also decreased ECM degradation, but did not substantially modify invadopodium formation. It is possible that the differences in these observations are due to the distinct experimental systems and/or to the presence/absence of the full-length protein. Although WIP binding to N-WASP is important for invadopodium formation^16^, little is known of the mechanisms by which it mediates this process. We demonstrate the importance of WIP in invadopodium assembly and describe its contribution to 3D invasion and BM degradation, which better mimic *in vivo* invasion than gelatin invasion assays^41^. Lack of WIP provoked no changes in overall cell morphology in Matrigel (Fig. 1), but significantly reduced cell invasive motility (Figs 1 and 2). WIP-deficient cells nonetheless showed less-stable protrusions in CIA (Fig. 1c,d) and altered paxillin recruitment to attachment structures (Fig. 1e,f), indicating a cell adhesion defect. Recent cell invasion research highlights a close relationship between FA and invadopodia. Not all cancer cells develop invadopodia to degrade and migrate into the matrix; some instead form FA with proteolytic activity^12^. FA and invadopodia share major components (vinculin, paxillin and β1-integrin) and regulatory pathways (FAK, Src, PI3K)^42^,^43^,^44^, and several reports propose that these structures are interconnected^10^,^45^. Our data agree with studies in dendritic cells, in which lack of WIP promoted instability of lamellar structures and altered focal contact turnover^46^. WIP regulation of invasive protrusions such as invadopodia (2D) and the pseudopods formed in Matrigel (3D) (Figs 1 and 2) as well as of FA stability^47^ (Fig. 1) suggests a related origin, and lends strength to a connection between invasive protrusions and FA. Further studies will clarify any association between FA and invasive protrusions, and elucidate the kinetics of components involved in early stages of invadopodium development.

Despite the biological relevance of 3D invasion experiments, we analyzed the regulation of invadopodium dynamics using gelatin invasion assays, given their robustness and consistency for examining protein expression and localization. Although the best-known WIP functions are dependent on its interaction with N-WASP, WIP also binds cortactin and Nck, which contribute respectively to initial and later steps in invadopodium development^11^,^14^,^16^,^48^,^49^,^50^,^51^. The WIP cortactin-binding domain, but not its Nck-binding domain, was needed to promote invadopodium formation in WIP-overexpressing MDA-MB-231 cells (Fig. 5). Alterations in persistence of movement reduce the migratory ability of Nck-depleted cells^52^, a phenotype remarkably like that of WIP-deficient cells, which suggests that these events are related. Nck recruitment and invadopodium initiation requires Src-dependent phosphorylation of cortactin residues Tyr421 or Tyr466^14^. Although WIP interacts with both proteins, its binding to Nck appears to be dispensable for promoting invadopodia (Fig. 5). We thus speculate that WIP is a necessary platform for indirect Nck recruitment, by direct binding to cortactin or through N-WASP interaction.

The effects of WIRE depletion were particularly evident in Matrigel experiments, in which the morphology of WIRE-depleted cells differed completely (shorter protrusions, rounded shape) from controls and WIP-deficient cells (Fig. 1g–i). N-WASP-deficient MDA-MB-231 cells show a phenotype similar to the WIRE-depleted cells, which is associated with a MT1-MMP trafficking defect that impairs matrix degradation^18^. Results in gelatin-invading cells support these findings, as WIRE-deficient cells formed immature invadopodia with decreased degradative ability (Fig. 2).

Similarities between WIRE and N-WASP deficiency effects, together with the wiskostatin treatment data (Fig. 4), indicate that N-WASP activity is WIRE-dependent in cancer cells. Reconstitution of WIRE-deficient cells with mutant WIP-ΔWBD was nonetheless successful, whereas cells that overexpressed mutant WIP-ΔNBD did not recover invasive ability (Fig. 5), which indicates that WIRE activation of N-WASP requires WIP-Nck binding. Both N-WASP-dependent^53^,^54^,^55^ and -independent roles^54^,^56^ have been attributed to WIRE. WIRE is involved in PDGF receptor endocytosis^54^; by regulating N-WASP-dependent endocytosis, it could participate in MT1-MMP recycling, leading to immature invadopodia.

WIP and WIRE are co-expressed in some cell types such as mouse embryonic fibroblasts^57^ and THP-1 monocytes (in which WIRE can bypass WIP deficiency and contribute to chemotaxis^58^). Here we show that WIP and WIRE are co-expressed in various BCC lines and control MDA-MB-231 cell invasion in different ways. In this model, neither endogenous nor exogenous WIRE expression was sufficient to rescue lack of WIP. In contrast, exogenous WIP overexpression (but not high endogenous WIP levels) rescued WIRE loss, which indicates a major WIP-exclusive function and suggests complementary functions for these two proteins. Exogenous WIP expression rescued invadopodium-mediated degradation in WIRE-deficient cells (Figs 3d and 5e,f), which indicates that WIP can adopt the WIRE cellular function and shows accurate regulation of both proteins. The finding that WIP overexpression significantly reduces WIRE levels and that WIRE depletion induces WIP overexpression (Fig. 3) supports this hypothesis. The failure of WIRE to rescue lack of WIP indicates that the domains shared by these proteins are insufficient to induce invadopodium formation; this supports the idea of a complex that regulates invadopodium initiation and assembly. WIP overexpression promoted invadopodium formation (Fig. 5c,d) and WIRE depletion promoted a significant increase in WIP (Fig. 3), which explains why a larger percentage of WIRE-deficient cells form invadopodia than controls (Fig. 2).

**Figure 8:**
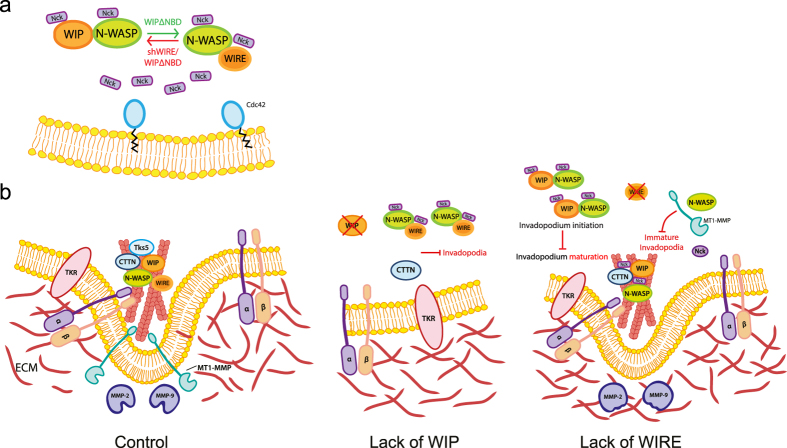
Model of the mechanisms that drive WIP- and WIRE-dependent ECM degradation. (**a**) Lack of WIP impairs invadopodium initiation, whereas mutant WIP-ΔNBD overexpression induces invadopodium formation and increases matrix degradation by active invadopodia, presumably by allowing free Nck binding to the N-WASP/WIRE complex. Conversely, when WIRE is reduced, cells develop immature invadopodia; when endogenous WIP and exogenous WIP-ΔNBD are present, WIP does not rescue the ability of invadopodia to degrade, as Nck might not bind N-WASP/WIRE. (**b**) In MDA-MB-231 control cells, a complex formed by cortactin, WIP and N-WASP facilitates invadopodium initiation and assembly by promoting actin polymerisation. Through WIRE activation, N-WASP mediates MT1-MMP transport to the membrane through F-actin binding, which gives rise to a mature invadopodium that degrades the ECM. In the absence of WIP, recruitment of N-WASP and cortactin is impaired, as is invadopodium assembly. In the absence of WIRE, N-WASP activity is diminished, which affects MT1-MMP traffic to the invadopodia, resulting in immature invadopodia unable to degrade the ECM.

Our results indicate that Nck contributes to WIP and WIRE functions during invasion. Whether Nck is recruited to invadopodia via direct interaction with WIP or as part of a multistep/multiprotein complex remains to be determined, but it appears to be critical for invadopodium-mediated invasion. Ditlev *et al*. proposed that N-WASP-Arp2/3 complex-mediated actin polymerization follows 4:2:1 Nck/N-WASP/Arp2/3 complex stoichiometry, in which two Nck molecules, one that binds WIP and the other N-WASP, interact in parallel with Arp2 and Arp3 subunits^31^. This concept allows us to hypothesize that an imbalance in Nck binding to WIP, WIRE and N-WASP could lead to distinct effects on actin polymerization and invadopodium function (Fig. 8). Invadopodia are functional in MDA-MB-231 cells, in which N-WASP binds WIP and WIRE; in WIP-lacking cells, which have fewer invadopodia, free Nck molecules might bind the N-WASP/WIRE complex. WIP-ΔNBD expression in WIRE-deficient cells did not rescue degradation, which indicates that Nck binding to this complex is essential for invadopodium maturation; in control cells, WIP-ΔNBD nonetheless promoted invadopodium-mediated degradation. These findings show that Nck-WIP binding is not necessary for invadopodium induction, but that lack of binding promotes their full degradative capacity, and suggests that Nck-WIP interaction negatively regulates invadopodium maturation. In the absence of WIRE, this equilibrium would be displaced towards the WIP/N-WASP complex (combined with WIRE depletion-induced WIP overexpression), which would promote Nck binding to WIP/N-WASP as well as development of invadopodia (not necessarily active, Fig. 8b).

Invadopodia have several discrete maturation stages, from precursor assembly to their development into a degradation-competent structure. Cortactin participates at the initial stage by recruiting other components, and at later stages when it must be dephosphorylated for consolidation of stable, long-lived protrusions^11^. Invadopodium maturation might require Nck liberation from cortactin, whose dephosphorylation prevents Nck binding. Compared to controls, WIRE-deficient invadopodium-like protrusions have less proteolytic capacity and concomitantly, decreased ability to allow Nck dispersion from their influence area (Fig. 6); this supports the hypothesis that Nck liberation is an obligate stage for complete invadopodium maturation. These findings offer a basis for further characterization of Nck, WIP and WIRE dynamics throughout invadopodium formation.

Previous mRNA array studies showed that WIP expression correlates with poor prognosis in various cancer types^36^,^59^. Several Oncomine datasets (https://www.oncomine.org/resource/login.html) indicate WIP overexpression in breast cancer samples (not shown; p < 0.05). Here we show significant WIP protein overexpression in highly invasive basal-B cells lines compared to less invasive basal-A and luminal cells (Fig. 7). This expression correlates with that of two other cytoskeletal proteins thought to be linked to breast cancer invasiveness, cortactin and fascin. Whereas cortactin data are debated^39^,^60^,^61^, several studies relate fascin expression to breast tumor aggressiveness and epithelial-to-mesenchymal transition^40^,^62^,^63^. Statistical analyses support association between high WIP or fascin expression and the basal-B invasive group; in our study, we found consistent high WIP expression in every basal-B cell line tested, which correlated with their invasive behavior *in vitro*^23^. Our findings for WIP protein confirm mRNA analyses^36^ and suggest WIP as a potential diagnostic marker. In addition to its role in cancer cell invasion, WIP is co-expressed with genes involved in proliferation and apoptosis^59^, which implies a major role in cancer progression by controlling distinct disease stages. The oncogenic properties of WIP, and its link to the actin cytoskeleton require further study.

That neither N-WASP nor WIRE expression correlated with invasiveness (Fig. 7), adds to the debate. Some studies report N-WASP overexpression at certain metastatic stages^18^,^64^ but another proposes that it is a tumor suppressor^65^. These discrepancies might be due to the nature of *in vitro* analysis, as expression of any of these proteins can differ at distinct cell stages, and requires validation in human tissue samples and at various metastatic stages. High WIP expression in invasive BCC and its potential to assume WIRE functions during invasion underlines the importance of WIP in breast cancer progression.

Based on WIP gene expression data^36^,^59^ and on our protein expression results, we propose WIP as a potential biomarker of breast cancer invasiveness. We also establish several mechanisms by which N-WASP, Nck, WIP and WIRE control cancer invasion. We propose that WIP is an essential factor for invadopodium assembly and that WIRE is an important element in maturation of invadopodia and invasive protrusions, and thus controls their ability to degrade the ECM.

## Materials and Methods

### Reagents and antibodies

Reagents included Dulbecco’s minimum essential medium (DMEM), fetal bovine serum (FBS) and gelatin from porcine skin (Type A, both from Sigma-Aldrich), GM6001 (Santa Cruz), wiskostatin (BIOMOL International), Alexa Fluor660 phalloidin and TO-PRO (Invitrogen) and Alexa Fluor488 phalloidin and DAPI (Sigma-Aldrich). We used monoclonal anti-cortactin (4F11, Millipore), -fascin (55K2, Abcam), -GAPDH (4G5, AbD Serotec), -Nck (BD Biosciences), -paxillin (Y113, Abcam) and polyclonal antibodies anti-WIP (H-224, Santa Cruz), -WICH/WIRE (HPA024467, Sigma-Aldrich), -N-WASP (H-100, Santa Cruz) and -collagen IV (Abcam). As secondary antibodies, we used Alexa Fluor405-goat anti-mouse IgG, Alexa488-goat anti-rabbit IgG, and Alexa647-goat anti-rabbit IgG (Invitrogen), HRP-goat anti-mouse IgG and HRP-goat anti-rabbit IgG (Santa Cruz).

### Cell culture, infection and transfection

Cell culture conditions are described in Table S1. Cells were infected using lentiviral stocks produced in 293T cells as described^23^,^66^. MDA-MB-231 cells were transfected with DNA plasmids using the Amaxa Nucleofector system (solution V, program X-013; Basel, Switzerland).

### DNA constructs, RNA interference

WIP and WIRE shRNA-coding plasmids were from Sigma (NM_003387 and NM_133264; MISSION). eGFP-fused full-length WIP and WIP deletion mutants (pLNT/Sffv-eGFP) are described^32^,^33^,^34^. Murine Flag-WIP and Flag-WIRE constructs were a gift of Dr Shigeru Tsuboi (Oyokyo Kidney Research Institute, Hirosaki, Japan).

### Time-lapse microscopy

For all time-lapse experiments, cells were incubated in a humidified 5% CO_2_ atmosphere at 37 °C. For time-lapse phase contrast imaging, we used a Nikon inverted fluorescence microscope TE 200 Wide Field fitted with a Nikon (Tokyo, Japan) Plan-Fluor 10x/0.3NA objective or a Leica inverted fluorescence microscope DMI6000B (Leica, Wetzlar, Germany) with a Leica HC PL 10 × 0.3NA objective lens and 1.6x magnification. For Nikon microscope imaging, we used a CoolSnap HQ2 charge-coupled device camera (Photometrics, Tucson, AZ) equipped with a Perfect Focus System device. The Leica microscope was fitted with a monochrome digital camera Orca R2, 12bit/16bit (Hamamatsu Photonics, Hamamatsu, Japan). Images were collected and processed with Metamorph v.7.7.7 (Molecular Devices, Sunnyvale, CA) or LAS AF v. 2.6.0 (Leica).

### Confocal microscopy

Confocal images were collected using the following inverted confocal microscopes: Bio-Rad Radiance 2100 confocal system in a Zeiss Axiovert 200 microscope (Zeiss, Jena, Germany) equipped with a Zeiss PlanNeoFluor 20x/0.5NA and Zeiss Plan-Apochromat 63x/1.4NA oil objective lens; Leica SP5 TCS (Leica, Mannheim, Germany) equipped with a Leica HCX PL Apo CS 40x/1.25NA oil objective lens and a Leica HCX PL Apo CS lambda blue 63x/1.4NA oil objective lens; and an Olympus Fluoview FV1000 confocal microscope (Olympus, Tokyo, Japan) equipped with an Olympus uPlan-SApochromat 60x/1.35NA oil objective lens.

### WIP gene expression analysis in breast cancer cells

Normalized microarray data of the WIP gene-specific probes 202664_at and 2026665_at were retrieved from two independent studies using 31 BCC lines^36^ and 51 BCC lines^35^. Cell lines were grouped according to molecular subtype (luminal, basal-A, basal-B/mesenchymal). Mean WIP expression was analyzed according to the *in vitro* invasive behavior of 28 BCC lines using the Neve dataset.

### Circular invasion assay

Experiments were performed as described^18^,^21^. Fluorescence intensity was quantified using ImageJ (US National Institutes of Health, http://imagej.nih.gov/ij/) for at least 15 cells per condition. Corrected total cell fluorescence (CTCF) was calculated as:


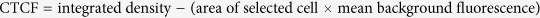


### Basement membrane invasion assay

Basement membranes (BM) were isolated from mouse peritoneum and prepared as described^18^,^28^,^29^. We seeded 2 × 10^5^ (for degradation experiments) or 5 × 10^4^ cells (for invadopodium formation).

### Inverse invasion assay

Inverted invasion assays were as described^18^,^23^,^67^.

### Fluorescent gelatin invasion assay

Fluorescent matrix-coated coverslips were prepared as described^68^. For wiskostatin treatment, cells were plated in medium supplemented with 10 μM GM6001 (to synchronize in-vadopodium formation) and 2 μM wiskostatin (BIOMOL International) or DMSO (adapted from^69^) (1 h, 37 °C). After extensive washing, cells were incubated with carrier or 2 μM wiskostatin-supplemented medium (3 h, 37 °C) before fixing. For each experiment, the number of invadopodium-forming cells (considering invadopodia as F-actin- and cortactin-positive ventral dots) and degrading cells (cells with invadopodia that overlap areas of degraded gelatin) was calculated by examining 25 random fields imaged with a 63x objective and represented after normalization to control values. Calculations of degraded area (degraded area/total cell area) were performed after subtracting a light background of 50 units and manually establishing a threshold to define degradation for each gelatin image file. Percentage of degraded area was normalized to the cell area. These measurements were made in at least 70 cells/condition from at least three independent experiments. Orthogonal views were generated from z-series with a 0.5 μm step size.

### Nck/actin/gelatin profiles

Localization of F-actin, Nck and gelatin were calculated by averaging fluorescence intensity over 10 pixels per profile and normalizing values to a scale from 0 to 1 in arbitrary units. Profiles were drawn crossing the whole cell and protein was localized at regions of interest (PM, plasma membrane, PN, perinuclear, IN, invadopodia). Raw data acquired for each image were exported in a numerical matrix format to OriginPro software (OriginLab). For each image, a perpendicular and horizontal profile was obtained, through invadopodia when present. A 2D area containing the intersection of these profiles was plotted as a surface contour map of normalized fluorescence intensity to determine the preferential distribution of each protein at the invadopodia.

### Immunoassays

IF and Western blot analysis were performed as described^23^. Densitometry analyses were performed using ImageJ.

### Statistical analysis

Statistical analyses were performed using Prism 5.0b for Mac OS × software (GraphPad Software).

## Additional Information

**How to cite this article**: García, E. *et al*. WIP and WICH/WIRE co-ordinately control invadopodium formation and maturation in human breast cancer cell invasion. *Sci. Rep.* **6**, 23590; doi: 10.1038/srep23590 (2016).

## Supplementary Material

Supplementary Information
