# Supplementary material for: WIP and WICH/WIRE co-ordinately control invadopodium formation and maturation in human breast cancer cell invasion
Source: Sci Rep. 2016 Mar 24;6:23590. doi: 10.1038/srep23590 (PMC4806363; doi:10.1038/srep23590)
Supplement: Supplementary Information [file srep23590-s1.pdf]

WIP and WICH/WIRE co-ordinately control invadopodium formation and maturation in human breast cancer cell invasion

Esther García, Chiara Ragazzini, Xinzi Yu, Elena Cuesta-García, Jorge Bernardino de la Serna, Tobias Zech, David Sarrió, Laura M. Machesky and Inés M. Antón

SUPPLEMENTARY INFORMATION

SUPPLEMENTAL TABLES

Table S1. Characteristics of BCC cell lines

Cell line	Gene cluster	ER/PR/HER2 expression	Tumor type	Culture medium
BT474	luminal	+ / + / +	IDC	RPMI, FBS, In, $NaHCO_3$
HS578T	basal B	- / - / -	IDC	DMEM, FBS, In
MCF7	luminal	+ / + / -	IDC	DMEM, FBS
MDA-MB-157	basal B	- / - / -	MC	DMEM, FBS
MDA-MB-231	basal B	- / - / -	AC	DMEM, FBS
T47D	luminal	+ / + / -	IDC	RPMI, FBS, SP, $NaHCO_3$
SKBR3	luminal	- / - / +	AC	McCoys 5A, FBS

Tumor type: IDC, invasive ductal carcinoma; F, fibrocystic disease; MC, metaplastic carcinoma; AC, adenocarcinoma. Culture conditions: RPMI, Roswell Park Memorial Institute 1640 medium; DMEM, Dulbecco's modified Eagle's medium (both from Sigma); F-12, Ham's F12 nutrient mixture (Gibco); FBS, fetal bovine serum 10%; HS, horse serum 5% (both from Sigma); HC, hydrocortisone 0.5 mg/ml (Calbiochem); EGF, epidermal growth factor 20 ng/ml (Peprotech); In, insulin (10 μg/ml, Gibco); $NaHCO_3$ 0.075% (Sigma); SP, sodium pyruvate (1 mM, Sigma)

SUPPLEMENTAL FIGURE LEGENDS

Figure S1. Expression of WIP family proteins in BCC and WIP- and WIRE-deficient cells. (a) Relationship between WIP mRNA levels and BCC invasive behavior (1). (b) WIP mRNA levels in basal-B, basal-A and luminal BCC (2). (c) MDA-MB-231 cells were transduced with lentiviral particles containing different shRNA-coding plasmids that target WIP or WIRE. Representative WB in which cell lysates (30 μg/lane) were examined using anti-WIP, -WIRE and -GAPDH antibodies. (d) Quantification of WIP and WIRE expression after depletion. Data shown as mean ± SD of at least three independent experiments. ** $p < 0.01$, *** $p < 0.001$ by 1-way ANOVA and Tukey's post-hoc test.

Figure S2. Disruption of Nck binding to WIP leads to opposite phenotypes in control and WIRE-deficient cells. Orthogonal views of cells shown in Figure 5. (a) MDA-MB-231 cells overexpressing WIP or WIPΔNBD (eGFP, green) were plated on rhodamine-gelatin (red, 5 h), fixed and stained for cortactin (cyan). (b) shWIRE cells overexpressing WIP, WIP-ΔCBD, WIP-ΔNBD or WIP-ΔWBD (eGFP, green). Arrows indicate invadopodia.

REFERENCES

1. Neve, R. M. et al. A collection of breast cancer cell lines for the study of functionally distinct cancer subtypes. *Cancer Cell* **10**, 515-527 (2006).
2. Charafe-Jauffret, E. et al. Gene expression profiling of breast cell lines identifies potential new basal markers. *Oncogene* **25**, 2273-2284 (2006).

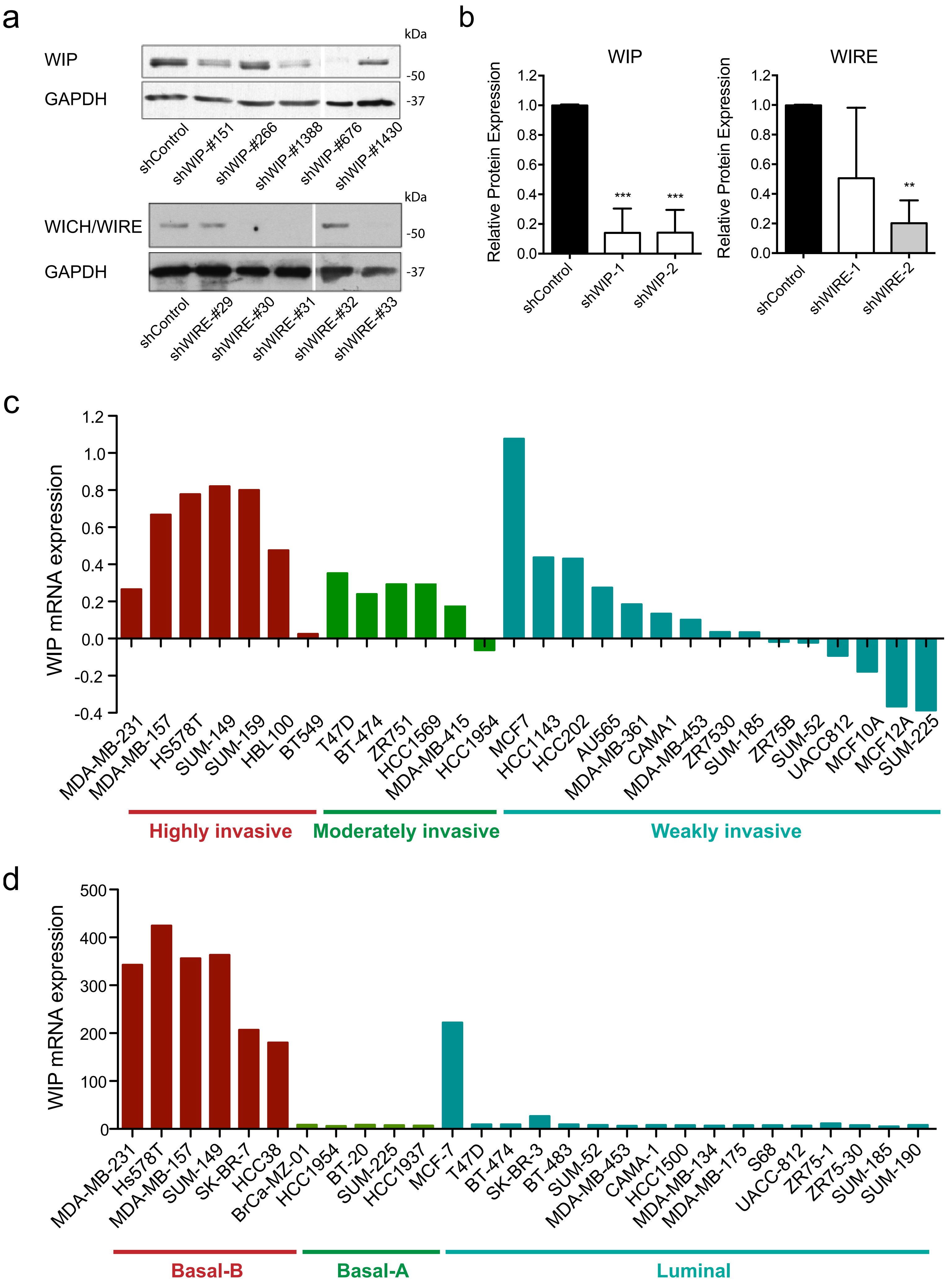
a
WIP
GAPDH
kDa
-50
-37
shControl
shWIP-#151
shWIP-#266
shWIP-#1388
shWIP-#676
shWIP-#1430
WICH/WIRE
GAPDH
kDa
-50
-37
shControl
shWIRE-#29
shWIRE-#30
shWIRE-#31
shWIRE-#32
shWIRE-#33
b
WIP
Relative Protein Expression
1.2
1.0
0.8
0.6
0.4
0.2
0.0
***
***
shControl
shWIP-1
shWIP-2
WIRE
Relative Protein Expression
**
shControl
shWIRE-1
shWIRE-2
c
WIP mRNA expression
1.2
1.0
0.8
0.6
0.4
0.2
0.0
-0.2
-0.4
MDA-MB-231
MDA-MB-157
HS578T
SUM-149
SUM-159
HBL100
BT549
T47D
BT-474
ZR751
HCC1569
MDA-MB-415
HCC1954
MCF7
HCC1143
HCC202
AU565
MDA-MB-361
CAMA1
MDA-MB-453
ZR7530
SUM-185
ZR75B
SUM-52
UACC812
MCF10A
MCF12A
SUM-225
Highly invasive
Moderately invasive
Weakly invasive
d
WIP mRNA expression
500
400
300
200
100
0
MDA-MB-231
Hs578T
MDA-MB-157
SUM-149
SK-BR-7
HCC38
BrCa-MZ-01
HCC1954
BT-20
SUM-225
HCC1937
MCF-7
T47D
BT-474
SK-BR-3
BT-483
SUM-52
MDA-MB-453
CAMA-1
HCC1500
MDA-MB-134
MDA-MB-175
S68
UACC-812
ZR75-1
ZR75-30
SUM-185
SUM-190
Basal-B
Basal-A
Luminal

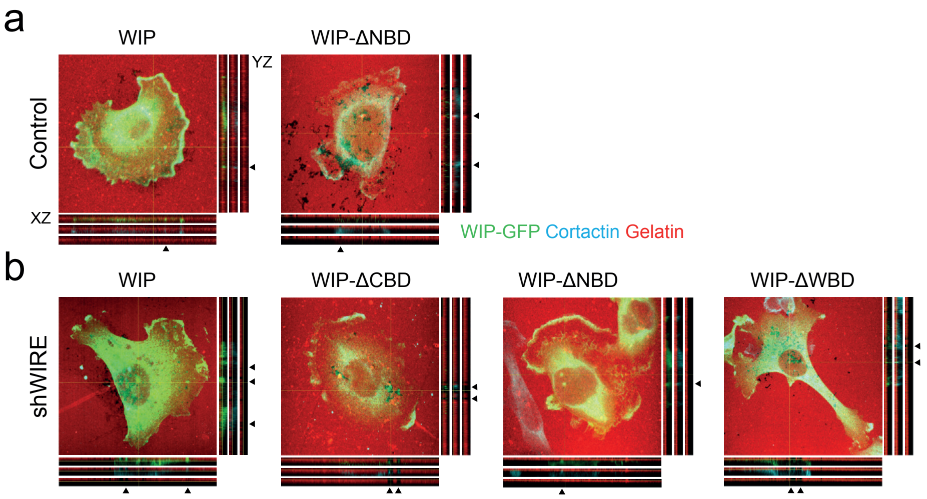
a
WIP
WIP-ΔNBD
Control
YZ
XZ
WIP-GFP Cortactin Gelatin
b
WIP
WIP-ΔCBD
WIP-ΔNBD
WIP-ΔWBD
shWIRE
